# Supplementary material for: Basement membrane proteins as a substrate for efficient Trypanosoma brucei differentiation in vitro
Source: PLoS Negl Trop Dis. 2021 Apr 28;15(4):e0009284. doi: 10.1371/journal.pntd.0009284 (PMC8109799; doi:10.1371/journal.pntd.0009284)
Supplement: Attachment — Submitted filename: Response to reviewers Rojas et al.docx [file pntd.0009284.s001.docx]

Thank you for sending on the referees’ comments on our original manuscript which all highlighted the exceptional interest and importance of our paper. We also are grateful for the referees’ valuable suggestions. In the revised submission (and our responses to the Referees’ comments) we have addressed all of the points raised. Specifically,

- Reviewer 2 requested we include a 0h time point for the experiment mapping stumpy to procyclic differentiation. We have now included this data point and also a procyclic cell-positive control.
- Reviewer 2 suggested we reduce the length of the manuscript to have a more focused discussion and to include whether our system is likely to allow in vitro investigation into quorum sensing in African trypanosomes. We have now reduced the length of the discussion section; the use of this in vitro system to investigate the quorum sensing signal in African trypanosomes was already discussed in the original manuscript.
- We have now included the number of cells counted for each experiment as requested by reviewer 1.

Point by point responses to reviewer’s comments:

**Methods**

Reviewer #1: In this manuscript the authors have set out to develop a robust in vitro system to support Trypanosoma brucei differentiation from slender to stumpy and then onto procyclic forms. The methodology of this paper is appropriate to address the objectives of this study. Moreover, this will be an incredible useful approach for the dissection of this differentiation process.

Reviewer #2: The study is well carried out and the statistically analyses and N numbers clearly explained.

We thank the Referees for this very clear statement on the importance and quality of our work and the expectation that this methodology will enable the study of this important differentiation process *in vitro*.

The PAD1 and EP pos/neg counting is done by microscopy scoring which is susceptible to bias. The authors should indicate whether the counting was done blinded or give more details on their criteria regarding thresholding etc.

Cells were considered PAD1 positive when expression of this stumpy marker was observed at the surface of parasites (slender cells do not express PAD1 and so are considered negative). Early during differentiation into stumpy forms, the PAD1 protein can be observed near the kinetoplast at the flagellar pocket region. These cells were considered as negative. We did not perform the counting blinded. These criteria have now been included in the methods section of the revised manuscript.

**Results**

Reviewer #2: The authors use tried and tested methods to determine the effect of BME upon slender-stumpy differentiation in culture. The results are unambiguous, well presented and support their conclusions. The dose response experiments (Figure 2A) are especially compelling.

The authors should extend this to determine whether increases in stumpy form differentiation efficiency levels out above a threshold BME concentration.

From the concentrations we tested, the amount the BME necessary to elicit an increase of cell differentiation is above 50 µl of BME (per 2ml of culture). The level of differentiation increases to at least 80% with 300µl (Figure 2A) but we have not gone further because the amount of BME would restrict the volume of HMI9 in the medium, potentially leading to nutrient limitation or other effects.

Can the authors comment on why the pleomorphic cells have similarly retarded growth in BME and methylcellulose and geltrex, but BME supports far higher PAD1 pos cells and cell cycle arrest? (Figure 1)

Although cells have similar growth profile in the different conditions tested, BME provides protein substrates that can generate the differentiation signal, inducing cell cycle arrest and expression of the stumpy marker PAD1. In contrast, HMI-9 and geltrex show limiting growth but differentiate less; we interpret this as being due to other factors that restrict growth without promoting the quorum sensing response and, in the case of geltrex, there being less substrate than in BME to drive quorum sensing (Figure 1b). In our hands, Methylcellulose allowed the cells to proliferate to higher densities compared with the other media tested- perhaps the physical characteristics of methylcellulose aid cytokinesis. However, this did not enhance differentiation.

Figures:
Figure 1: growth curve should be log scale (and others throughout the manuscript)

We believe that having the growth profile shown on a linear scale communicates the effects in a more intuitive way than on a logarithmic scale. For example, Methylcellulose allows cells to grow to higher densities than any other condition tested and by converting to log scale, this effect is less obvious.

Figure 2: a 0hrs timepoint should be included

We have included as the reviewer suggested the 0h time point for the procyclic differentiation and a positive control (procyclic cells) in Figure 2b.

**Conclusions**

Reviewer #1: Overall, the data in the paper supports the conclusion and the graphs and images are clear and well presented. The authors are able to show that the addition of BME to the media supports differentiation of pleomorphic trypanosomes through the quorum-sensing pathway.

Reviewer #2: The authors’ conclusions are supported by the data – i.e. BME supports development of stumpy forms better than other media additives and it is likely to be a good method for studying differentiation in trypanosomes.

A discussion on whether this system is likely to allow in vitro investigation into quorum sensing in related African trypanosomes would be worthwhile.

In the original submitted paper, we discussed that the use of BME will help in the characterization of the quorum sensing signal in *Trypanosoma brucei* (line 459). We have not explored effects in related trypanosomes (e.g. T. congolense); these parasites grow in different media and do not have transmission stages identifiable using existing markers. Nonetheless we expect the in vitro system to assist with the characterisation of differentiation particularly in *T. brucei* and, potentially, in other trypanosome species, and this work is underway in our laboratory.

**Editorial and Data Presentation Modifications**

Reviewer #1: Can the authors provide the number of cells counted and the replicates performed for each experiment in the figure legend – some of this information is in the methods but not all and I find it simpler if this is included with the figures.

We have now included the number of cells counted and replicates in each experiment in the figure legends.

2) How were the growth analyses performed – can the authors add detail. Are these cumulative growth curves? Were the cells split each day?

The growth analyses were started on day 0 and cells were allowed to grow for 72hs without passage for the duration of the experiment. This methodology allows the cells to receive the differentiation signal, if present, and to assess how the culture media contributes to slender to stumpy differentiation.

3) The balance of the paper seems odd there are only 5 pages of results which includes the figure legends yet there is also 5 pages of discussion – this seems excessive. In the discussion there is lots of detail about collagen (as there was in the introduction) and various peptides that can form and their half-lives etc. I feel this though interesting would be more appropriate for a review of this topic rather than as a discussion of this paper, which is essentially a technology development paper. I would recommend that the authors reduce the length of the discussion to give it greater focus.

We thank the reviewer’s suggestion. We have now reduced the length of the discussion by eliminating the excess of information on collagen and peptide half-life. This section is more focused now on the discussion of the results we obtained and the possibilities that this system offers, not only for the investigation of the differentiation process in trypanosomes, but also to in determine the exact components of the quorum sensing signal generated by these parasites.

**Summary and General Comments**

Reviewer #1: (No Response)

Reviewer #2: This study demonstrates that BME can act as a media supplement that can support the efficient differentiation from slender to stumpy forms in pleomorphic Trypanosoma brucei. The authors use various assays (cell cycle arrest, stumpy molecular markers and lifecycle progression) to show this successfully.

This is a useful methods paper that will facilitate / promote the study of slender-stumpy form differentiation by providing a superior in vitro culture system. The advantages of such as system are two-fold: 1) This system has the potential to be far more tractable than in vivo infection. 2) this will reduce the number of mice that must be sacrificed.

This will be of interest to those studying trypanosome differentiation.

Again, we thank the reviewer for highlighting the usefulness of this methodology for studying an important differentiation process in the life cycle of *Trypanosoma brucei*, and the benefits of using a tractable in vitro system that will benefit the whole parasitology community.
